# Synthesis and Characterization of Thallium-Texaphyrin Nanoparticles and Their Assessment as Potential Delivery Systems for Auger Electron-Emitting ^201^Tl to Cancer Cells

**DOI:** 10.1021/acs.molpharmaceut.4c00873

**Published:** 2024-12-16

**Authors:** Katarzyna M. Wulfmeier, Miffy H. Y. Cheng, Zhongli Cai, Samantha Y. A. Terry, Vincenzo Abbate, Philip J. Blower, Gang Zheng, Raymond M. Reilly

**Affiliations:** † Department of Imaging Chemistry and Biology, School of Biomedical Engineering and Imaging Sciences, King’s College London, London SE1 7EH, U.K.; ‡ Princess Margaret Cancer Centre, University Health Network, Toronto M5G 1L7, Canada; § Department of Pharmaceutical Sciences, Leslie Dan Faculty of Pharmacy, University of Toronto, Toronto M5S 1A1, Canada; ∥ Institute of Pharmaceutical Sciences, King’s College London, London SE19NH, U.K.; ⊥ Department of Medical Biophysics, University of Toronto, Toronto M5G 1L7, Canada; # Department of Medical Imaging, University of Toronto, Toronto M5S 1A8, Canada

**Keywords:** ^201^Tl, Auger electrons, texaphyrin-lipid, targeted radionuclide therapy, radiation nanomedicines

## Abstract

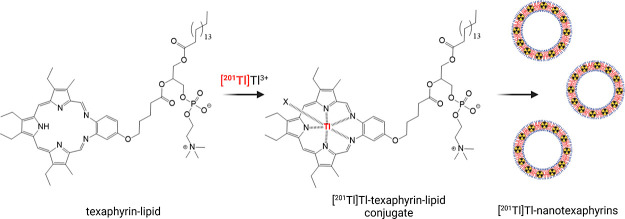

Thallium-201 is an Auger electron-emitting radionuclide with significant potential for targeted molecular radiotherapy of cancer. It stands out among other Auger electron emitters by releasing approximately 37 Auger and Coster–Kronig electrons per decay, which is one of the highest numbers in its category. It has also a convenient half-life of 73 h, a stable daughter product, established production methods, and demonstrated high *in vitro* radiotoxicity. However, its full potential in targeted radiotherapy remains unexplored, primarily due to the lack of available efficient chelators for [^201^Tl]Tl^+^ or [^201^Tl]Tl^3+^. This study aims to assess texaphyrin for macrocycle chelation of [^201^Tl]Tl^3+^. Texaphyrins are known for effective binding of trivalent metals with similar ionic radii, such as indium and gadolinium. Optimization of [^201^Tl]Tl^+^ to [^201^Tl]Tl^3+^ oxidation and subsequent chelation with texaphyrin-lipid conjugate were assessed using thin-layer chromatography. The formation and stability of nonradioactive Tl-texaphyrin-lipid complexes were confirmed by UV–Vis spectroscopy and ultrahigh performance liquid chromatography–mass spectrometry. [^201^Tl]Tl/Tl-texaphyrin-lipid nanoparticles (nanotexaphyrins) were assembled by using a microfluidic system, and their morphology and stability were evaluated by using dynamic light scattering and transmission electron microscopy. The uptake of these nanotexaphyrins in lung cancer and ovarian cancer cells was evaluated using both radioactive and nonradioactive methods. The conversion of [^201^Tl]Tl^+^ to [^201^Tl]Tl^3+^ in 0.25 M HCl achieved an average yield of 91.8 ± 3.1%, while the highest radiolabeling yield of the texaphyrin-lipid with [^201^Tl]Tl^3+^ was 25.5 ± 4.5%. Tl-texaphyrin-lipid conjugates were stable at room temperature for at least 72 h. These conjugates were successfully assembled into homogeneous nanotexaphyrins with an average hydrodynamic diameter of 147.4 ± 1.4 nm. Throughout a 72 h period, no changes in size or polydispersity of the synthesized nanoparticles were observed. [^201^Tl]Tl-nanotexaphyrins were synthesized with an average radiochemical purity of 77.4 ± 10.3% and a yield of 5.1 ± 4.4%. The release of [^201^Tl]Tl^+^ from [^201^Tl]Tl-nanotexaphyrins in phosphate-buffered saline exhibited a time- and temperature-dependent pattern, with a faster release observed at 37 °C than at room temperature. Additionally, the uptake of Tl-nanotexaphyrins and [^201^Tl]Tl-nanotexaphyrins in cancer cells was similar to that of unbound Tl^+^ and [^201^Tl]Tl^+^. This is the first time that texaphyrins have been investigated as chelators for radiothallium. Although [^201^Tl]Tl-nanotexaphyrins were found to be thermodynamically and kinetically unstable, we successfully synthesized stable texaphyrin-lipid complexes with ^nat^Tl^3+^. This opens up opportunities for further refinements in the nanotexaphyrin-lipid structure to enhance [^201^Tl]Tl^3+^ stability and prevent its reduction to a 1+ oxidation state. Future research should consider further modifications to the texaphyrin structure or using texaphyrins without the lipid component.

## Introduction

Among the extensive group of Auger electron emitters, ^201^Tl has been identified as one of the most promising radionuclides for targeted therapy of cancer, based on its physicochemical properties and favorable cellular and multicellular dosimetry profiles.^1^^201^Tl has one of the highest numbers of Auger electrons (AE) and Coster–Kronig (CK) electrons emitted per decay (average 36.9 electrons/decay).^2^ The short range of these electrons leads to a high linear energy transfer, typically between 1 and 23 keV/μm, and the potential to induce clustered damage if targeted to the cell nucleus or the cell membrane.^3^ It has been previously demonstrated that ^201^Tl is highly radiotoxic, capable of triggering multiple nuclear double-strand DNA breaks and significant cytotoxicity against prostate and breast cancer cells but only when internalized.^4^ Furthermore, ^201^Tl possesses a convenient half-life (73 h), a stable daughter (^201^Hg), and an established production method due to its previous extensive use in myocardial perfusion scintigraphy. These characteristics make it a promising candidate for precise targeted cancer radiotherapies aimed at small tumors and micrometastases. However, this clinical application of ^201^Tl is impeded by the lack of efficient chelating agents available for complexing it with targeting molecules.

^201^Tl is mostly available as Tl^+^ (Tl(I)) in aqueous solutions, and strong oxidation conditions are needed to generate Tl^3+^ (*E*° = +1.25 V).^5,6^ Tl^3+^ has a much smaller ionic radius in comparison to Tl^+^ (103 pm vs 164 pm, for six-coordinate ions) and chemically resembles gallium and indium, except for its facile reduction to Tl(I).^7^ Only a few chelators for Tl^3+^ have been assessed in the past, for example, tetraazacyclododecanetetraacetic acid (DOTA),^7^ diethylenetriaminepentaacetic acid (DTPA),^6^ ethylenediaminetetraacetic acid (EDTA),^5^ H_4_pypa,^8^ and porphyrin derivatives,^9^ but all proved to form complexes with low stability when tested either *in vitro* or *in vivo*, likely due to the facile reduction of Tl^3+^ to a 1+ oxidation state with subsequent or simultaneous metal dissociation from the metal complex. Bis-thallium(I) complexes of porphyrin derivatives using octaethylporphyrin (OEP) and tetraphenylporphyrin (TPP) were prone to metal loss in solution.^10^ Tl(III)-OEP-Cl crystal structure showed a 5-coordinate complex with the central Tl^3+^ ion displaced above the plane of the porphyrin ring causing a distortion in the porphyrin macrocycle.^11,12^*In vivo* tests with 5,10,15,20-tetrakis(pentafluorophenyl)porphyrin (PFPP)-Tl(III) complex in healthy rats revealed poor chelation stability, leading to free thallium ion accumulation in the myocardium.^13^ Tl^3+^ and Tl^+^ ions, while capable of forming complexes with porphyrin derivatives, both appeared too large for the central porphyrin macrocycle, resulting in geometric distortion and instability.^11^

Texaphyrins are a synthetic subclass of porphyrins, with a larger pentadentate cavity that binds metals through five donor nitrogen atoms, compared to a tetradentate cavity with four donor nitrogen atoms offered by porphyrins (Suppl. Figure 1).^14^ The inner coordination core of texaphyrins is around 20% larger in radius than the core present in porphyrin structures. The larger texaphyrin ring enables the formation of stable 1:1 complexes with a variety of bi- and trivalent metals, including indium (In^3+^), bismuth (Bi^3+^), manganese (Mn^2+^), and lanthanides.^14^ The molecular structure, stability, charge distribution, and electron affinity of actinide(III) complexes with motexafins, which are more stable derivatives of texaphyrins, have been evaluated using density functional theory models.^15,16^ This computational analysis suggests that actinide(III) cations, particularly Ac^3+^, form tightly bound, stable complexes with the pentaaza texaphyrin macrocycle, highlighting the potential use of motexafins as chelators for ^225^Ac in alpha-emitter radiotherapy. An experimental approach was undertaken to chelate Bi^3+^ in aqueous conditions to optimize chelation kinetics and obtain stable texaphyrin complexes with ^213^Bi, an alpha-emitting radionuclide with potential in the treatment of micrometastatic tumor disease.^17^ The texaphyrin macrocycle can be further functionalized for various biomedical applications (Suppl. Figure 1),^18^ including conjugating polyethylene glycol (PEG) groups to increase texaphyrin bioavailability,^19^ attaching platinum-based compounds to overcome platinum resistance in cancer treatment,^20^ and incorporating a prostate cancer targeting ligand (prostate-specific membrane antigen, PSMA) into the texaphyrin structure for selective tumor delivery and enhanced cellular uptake.^21^ Recently, texaphyrin-lipid conjugates and their self-assembly into liposome-like nanoparticles, known as nanotexaphyrins, have been developed.^18^ The biomedical utility of nanotexaphyrins has been exemplified through the chelation of various metals to enable magnetic resonance imaging (MRI), single-photon emission computed tomography (SPECT), radionuclide therapy, and photodynamic therapy (PDT).^21^ The mix-and-match approach of diagnostic and therapeutic radionuclides has led to three classes of multimodal nanotexaphyrins, such as the dual diagnostic ^111^In/Mn nanotexaphyrin (SPECT/CT and MRI),^22^ the prostate-specific membrane antigen targeted radiotheranostics PSMA-^111^In/Lu nanotexaphyrin (tumor targeting, SPECT/CT and PDT),^21^ and myoglobin-loaded ^177^Lu/Gd-nanotexaphyrins (radiosensitizer, SPECT/CT and MRI).^23^ These studies had demonstrated the versatility of nanotexaphyrins and their potential applications as cancer nanotheranostic agents. Since nanotexaphyrins effectively bind radiometals and form stable complexes, texaphyrins can be proposed as feasible chelators for ^201^Tl, providing protection against environmental factors such as reducing agents by forming stable Tl^3+^-texaphyrin complexes.

In this study, we aimed to synthesize [^201^Tl]Tl-texaphyrin nanoparticles for the first time and to explore their potential as a novel delivery system for [^201^Tl]Tl^+^ and [^201^Tl]Tl^3+^ ions. We investigated the chelation capability of texaphyrin-lipids toward Tl^3+^ ions, assessed their stability, and validated the radiolabeling efficiency toward ^201^Tl. We characterized the self-assembled stable ^nat^Tl and radioactive [^201^Tl]Tl-nanotexaphyrin for its colloidal properties. Lastly, we tested these particles in cancer cells for radionuclide therapy purposes.

## Materials and Methods

### Materials

Thallous chloride solution (10 mM) was prepared by dissolving 2.4 mg of TlCl in 1000 μL of Milli-Q water (18.2 MΩ cm), sonicating, and heating at 40 °C for 1 h. HCl 0.05–2.5 M solution was prepared by diluting 37% HCl with Milli-Q water. Texaphyrin-lipids were synthesized according to the method described previously,^22^ and the structure was confirmed by ultrahigh performance liquid chromatography–mass spectrometry (UHPLC-MS). Texaphyrin-lipid solution (1 mM) was obtained by dissolving 1 mg in 920 μL of 99.9% ethanol. KCl solution (500 mM) was prepared by dissolving 1.86 g of KCl (EMD Millipore) in 50 mL of Milli-Q water. Cell culture consumables and chemical compounds, unless specified, were purchased from Sigma-Aldrich, Canada. [^201^Tl]TlCl in sterile, 0.9% NaCl solution (213–236 MBq/2.8 mL) was obtained from Curium, USA (2.8 mCi/2.8 mL on the calibration date). **Cell culture:** Human lung cancer cells A549 and human ovarian cancer cells SKOV3 were cultured in RPMI-1640 medium (R8758). High, nonspecific uptake of various types of nanoparticles by these cell lines has been previously described in the literature.^24,25^ Medium was supplemented with 10% fetal bovine serum (Gibco, 12484–028), penicillin (100 units), and 100 μg/mL streptomycin. Cultured cells were trypsinized and seeded at 100,000 cells per well in 24-well plates 16 h before each experiment and grown at 37 °C in a humidified 5% CO_2_ atmosphere. Cells were counted by using a Bio Rad T20 automated cell counter. **[**^**201**^**Tl]Tl**^**+**^**/Tl**^**3+**^**and**^**nat**^**Tl**^**+**^**/Tl**^**3+**^**oxidation:** The oxidation method described previously^5^ was used with some modifications. Briefly, 100 μL of [^201^Tl]TlCl (5.2–8.3 MBq) or stable 10 mM TlCl aqueous solution was added to an oxidation bead (Pierce Iodination Beads, N-chloro-benzenesulfonamide sodium salt, ThermoFisher Scientific), then 10 μL of 2.5 M HCl (Fisher Chemicals) was added, and the solution was shaken for 1 to 2 h. ^201^Tl oxidation efficiency was assessed by the instant thin layer chromatography (iTLC) method with acetone as a mobile phase and iTLC-SG chromatography paper (silica gel impregnated glass fiber, Pall Corporation) as a stationary phase (Suppl. Figure 2a). iTLC strips were imaged with a Cyclone Plus Storage Phosphor System. **[**^**201**^**Tl]Tl**^**3+**^**/**^**nat**^**Tl**^**3+**^**chelation with texaphyrin-lipids:** 100 μL of oxidized [^201^Tl]TlCl_3_ or TlCl_3_ was diluted 5-fold with 0.1 M NH_4_OAc buffer (pH = 7) or ddH_2_O, and 1 mM texaphyrin-lipid ethanol solution was added in volume matching the calculated molar ratio. For ^201^Tl, the molar ratio of texaphyrin-lipid:Tl was calculated as 1:0.005 based on the concentration of nonradioactive ^203^Tl in [^201^Tl]TlCl measured previously (0.8 μmol/L ^203^Tl).^4^ For nonradioactive thallium chelation, the texaphyrin-lipid:Tl ratio was set as 1:1 or 1:2. After adding [^201^Tl]TlCl_3_ or TlCl_3_ to the texaphyrin-lipid solution, the mixture was left for 1 h at room temperature (RT) to allow for chelation; a change in color from brown to green was observed when forming nonradioactive Tl-texaphyrin-lipids. An iTLC method involving the iTLC-SG chromatography paper and a mobile phase of 10% EDTA solution in 0.1 M NH_4_OAc was used to assess chelation efficiency of [^201^Tl]Tl-texaphyrin-lipids (Suppl. Figure 2b). iTLC strips were imaged with a Cyclone Plus Storage Phosphor System. UV–Vis spectra of nonradioactive Tl-texaphyrin-lipids were obtained with a Biochrom Ultraspec 3100 Pro spectrophotometer after 6- to 7-fold dilution in 99.9% ethanol. UHPLC-MS was performed using an Acquity UPLC CSH Phenyl-Hexyl 1.7 μm column (Waters Canada, Ontario, Canada) with a Waters 2695 controller, 2996 photodiode array detector, and a Waters triple quadrupole (TQ) mass detector (mobile phase: 0.1% TFA (A) and acetonitrile (B) in gradient (60% A + 40% B to 0% A + 100% B in 3 min, kept at 100% B for 1 min, followed by a drop to 60% A + 40% B, column temperature: 60 °C; flow rate: 0.6 mL/min; positive ion mode ESI). Samples were diluted 10-fold in 99.9% ethanol. Mn and Cd complexes with texaphyrin-lipids were synthesized, as described before.^18,20^**Synthesis of [**^**201**^**Tl]Tl-nanotexaphyrins and**^**nat**^**Tl-nanotexaphyrins:** 150 μL of ethanol solution containing cholesterol (CHL, 40.6% total molar lipid content), DSPE-PEG_2000_ (distearoylphosphatidylcholine polyethylene glycol 2000; 5.0% total molar lipid content), and 1 mg DPPC (dipalmitoylphosphatidylcholine, 34.4–44.4% total molar lipid content) (Avanti Polar Lipids, Inc.) was added to 100 μL [^201^Tl]Tl-texaphyrin-lipids or Tl-texaphyrin-lipids, mixed, and transferred to a 1 mL syringe and then slowly coinjected with 750 μL of 0.1 M NH_4_OAc buffer placed in a second 1 mL syringe via a microfluidic chip (Microfluidic Chip Shop). Crude [^201^Tl]Tl-nanotexaphyrin and Tl-nanotexaphyrin were purified with Amicon-Ultra 0.5 mL ultrafiltration devices (30,000 MWCO, Millipore), centrifuged for 10 min at 12,000 rpm, and then washed with 200 μL of phosphate-buffered saline (PBS) 3–5 times. An iTLC method (iTLC-SG chromatography paper, mobile phase: 10% EDTA solution in 0.1 M NH_4_OAc) was used to assess [^201^Tl]Tl-nanotexaphyrin purity (Suppl. Figure 2b). Activity of purified [^201^Tl]Tl-nanotexaphyrins was measured with a dose calibrator (Capintec) and compared to the starting [^201^Tl]TlCl activity for assessing the radiolabeling efficiency (RE). **Stability studies:** Tl-texaphyrin-lipids were kept for up to 72 h either at room temperature or 37 °C in ethanol solution and analyzed visually, by UV–Vis spectrometry and UHPLC-MS chromatography, as described above. The size and polydispersity of synthesized Tl-nanotexaphyrins were assessed in PBS by dynamic light scattering (DLS) measurements over a period of 72 h in a temperature range of 4–37 °C. [^201^Tl]Tl-nanotexaphyrins were kept in PBS at 4 and 37 °C for up to 72 h, and the radiostability was tested with an iTLC method (stationary phase: silica gel impregnated glass fiber; mobile phase used: 10% EDTA in 0.1 M NH_4_OAc). **Characterization of**^**nat**^**Tl-nanotexaphyrins:** To determine the hydrodynamic diameter, polydispersity index (PDI), and surface charge of the nanoparticles, Tl-nanotexaphyrins were characterized using a Malvern Zetasizer Nano-ZS 90 device. The Tl-nanotexaphyrin suspension was diluted 10 times with PBS for the size measurements, whereas no dilutions were made for the zeta potential measurements. Transmission electron microscopy (TEM) images were obtained with an FEI Tecnai 20 microscope (200 kV voltage). Tl-nanotexaphyrin suspension was placed on a carbon-coated copper grid and negatively stained with 2% uranyl acetate. **Cellular uptake:** A549 or SKOV3 cells were seeded at 100,000 cells per well in 24-well plates, as described above. Fifteen minutes before the experiment, culture medium was replaced by 190 μL (for cells incubated with KCl) or 200 μL of fresh RPMI medium. To assess the impact of K^+^ on [^201^Tl]Tl/Tl-nanotexaphyrins uptake, cells were also incubated with 25 mM KCl as a final concentration (10 μL of 500 mM KCl solution was added to 190 μL of medium, followed by 50 μL of either [^201^Tl]Tl/^nat^Tl^+^ or [^201^Tl]Tl/Tl-nanotexaphyrins). Purified [^201^Tl]Tl-nanotexaphyrins or Tl-nanotexaphyrins were diluted with PBS to the required concentration (1 MBq/mL or 20 μmol/L), and 50 μL was added to each well. [^201^Tl]TlCl or TlCl was oxidized by the method described above and diluted with PBS to the same concentration as [^201^Tl]Tl/Tl-nanotexaphyrins, and 50 μL was added to each well. Plates were incubated at 37 °C for 16 h, and then, the incubation solution was collected. Adherent cells were briefly washed 3 times with PBS and lysed with 1 M NaOH for 15 min at room temperature. To determine the radioactive uptake, unbound radioactivity (incubation medium and PBS washings) and cell-bound radioactivity (lysate) were measured (counts per minute, CPM) with a gamma counter (PerkinElmer Wallac Wizard 3 1480 Automatic Gamma counter). For nonradioactive uptake studies, unbound thallium (medium and PBS washings) and cell-bound thallium (lysate) samples were placed in a vacuum centrifuge and left overnight to evaporate water. Then, 100 μL of 70% (v/v) HNO_3_ (environmental grade) was added to each Eppendorf tube and heated at 60 °C for 2 h, and 1000 μL of 2% (v/v) HNO_3_ was added to each sample, the contents vortexed and centrifuged for 5 min at 12,000 rpm. 800 μL of the supernatant was collected, added to 2 mL 2% (v/v) HNO_3_, mixed, and analyzed by the PerkinElmer NexION 350 inductively coupled plasma-mass spectrometer. A series of standards (10^–2^–10^–7^ mg/L) were prepared by diluting 1 g/L TlCl solution in 2% (v/v) HNO_3_, and linear regression lines were fitted for ^203^Tl and ^205^Tl. The quality of the inductively coupled plasma-mass spectrometry (ICP-MS) measurements was ensured through repeated measurements of blanks and internal ^193^Ir standards. **Cell fractionation:** A fractionation method was used whereby 15 min before the experiment, medium in each well containing A549 or SKOV3 cells were replaced by 200 μL fresh medium.^26^ Purified [^201^Tl]Tl-nanotexaphyrins were diluted with PBS to the required concentration (1 MBq/mL), and 50 μL was added to each well. Oxidized [^201^Tl]TlCl_3_ was diluted in the same manner. After 16 h of incubation at 37 °C in a humidified 5% CO_2_ atmosphere, the 24-well plates were put on ice, the radioactive incubation solution containing unbound ^201^Tl was collected, and adherent cells were briefly washed three times with 250 μL of ice-cold PBS. The activity present in all PBS washes and the medium was measured with a gamma counter (“medium” fraction). Then, 250 μL of ice-cold 200 mM sodium acetate solution and 500 mM sodium chloride (pH = 2.5) were added to each well to remove cell surface bound ^201^Tl, incubated with cells on ice for 10 min and then removed, combined with a subsequent wash of 250 μL of cold PBS, and measured as a fraction using the gamma counter (“membrane” fraction). Next, the cells were lysed with 250 μL of cold Nuclei EZ Lysis buffer on ice to isolate internalized ^201^Tl. After 2 h incubation, cells were transferred to Eppendorf microcentrifuge tubes, centrifuged for 5 min at 1000 rpm, and the supernatant was collected. The remaining pellet was resuspended in 250 μL of cold PBS, centrifuged, and again the supernatant was collected. This process was repeated, and the supernatants were combined. The activity present in the combined supernatant fractions (“cytoplasmic” fraction) and the activity present in the pellet (“nuclear” fraction) were quantified with a gamma counter (as CPM). **Data analysis:** The results were analyzed in Excel Microsoft 2016, GraphPad Prism 9, and ChemDraw 20.1 and expressed as mean ± SD. To assess the significance, the paired *t* test was used and the value of *P* < 0.05 was considered statistically significant. Figures were created with GraphPad Prism 9, BioRender.com, and ChemDraw.

## Results

### Oxidation of [^201^Tl]Tl^+^ to [^201^Tl]Tl^3+^ prior to Chelation

**Figure 1:**
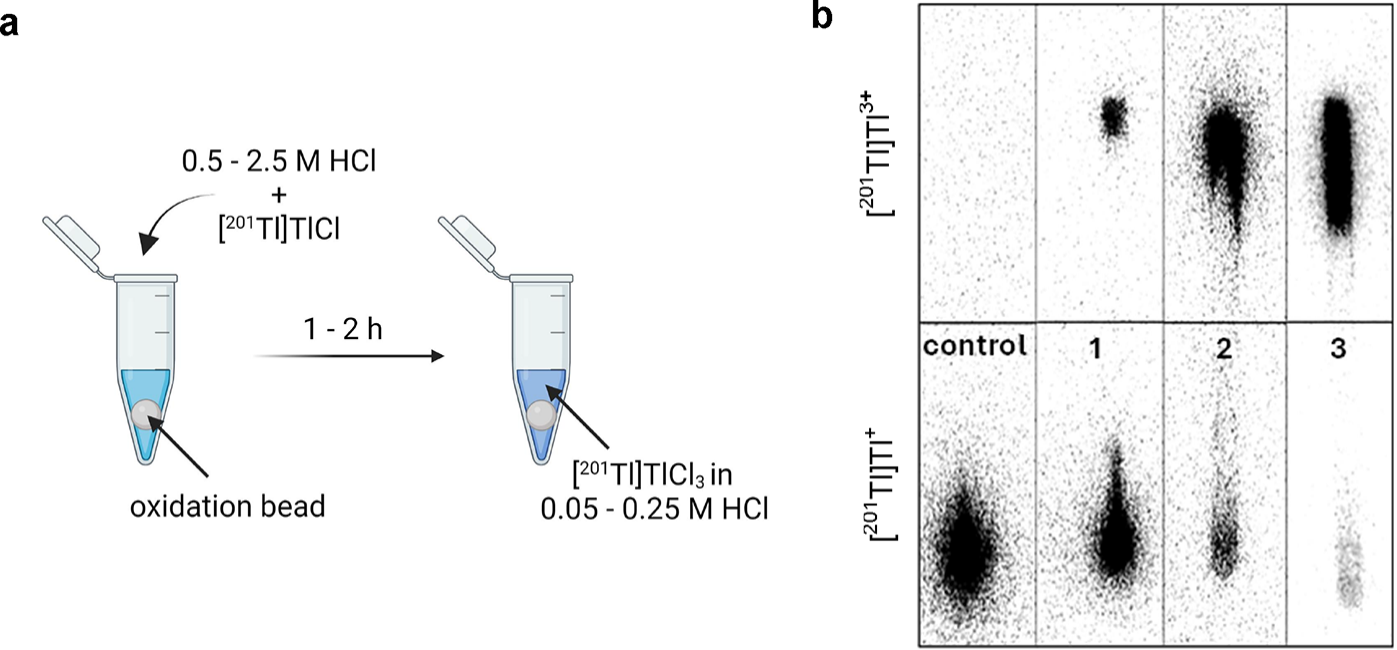
Oxidation of [^201^Tl]Tl^+^to [^201^Tl]Tl^3+^. (a) Schematic illustration of conversion of [^201^Tl]Tl^+^to [^201^Tl]Tl^3+^ performed with an oxidation bead (iodination bead). (b) iTLC analysis of [^201^Tl]Tl^+^to [^201^Tl]Tl^3+^oxidation reaction in various concentrations of HCl imaged with a phosphor imager. iTLC strip representing [^201^Tl]Tl^+^ at the origin (bottom) when no oxidation agent was used (control); iTLC strips representing [^201^Tl]Tl^+^ at the origin (bottom) and [^201^Tl]Tl^3+^ at the solvent front (top) after oxidation reaction was performed in 0.05 M HCl solution (1), 0.1 M HCl solution (2), and 0.25 M HCl solution (3). Stationary phase: iTLC-SG; mobile phase: acetone. [^201^Tl]Tl^3+^ in these conditions migrates with the solvent front while [^201^Tl]Tl^+^ remains at the origin.

First, we investigated the oxidation of [^201^Tl]TlCl using a solid-phase N-chloro-benzenesulfonamide (Iodination/oxidation beads) under different molar concentrations of HCl since the addition of the appropriate HCl concentration proved crucial for oxidation reaction yield (Figure 1a). The oxidation of [^201^Tl]Tl^+^to [^201^Tl]Tl^3+^ was quantified by the iTLC method (using acetone as the mobile phase, Suppl. Figure 2a). Increasing HCl concentration from 0.05 to 0.1 and 0.25 M enhanced the conversion of [^201^Tl]Tl^+^ to [^201^Tl]Tl^3+^ (Figure 1b). The iTLC also revealed that 0.25 M HCl resulted in an average of 91.8 ± 3.1% of [^201^Tl]Tl^3+^ converted from [^201^Tl]Tl^+^. Following the removal of the oxidation bead from the reaction mixture, the oxidized [^201^Tl]Tl^3+^ remained stable for at least 72 h when stored at 4 °C in 0.25 M HCl (Suppl. Table 1). Despite experiencing some losses in activity during extraction from the oxidation beads, we were still able to recover more than 60% of radioactivity for radiolabeling of texaphyrin-lipid.

### Nonradioactive Tl^+^/Tl^3+^ and Radioactive [^201^Tl]Tl^+^/[^201^Tl]Tl^3+^ Chelation by Texaphyrin-Lipids

**Figure 2:**
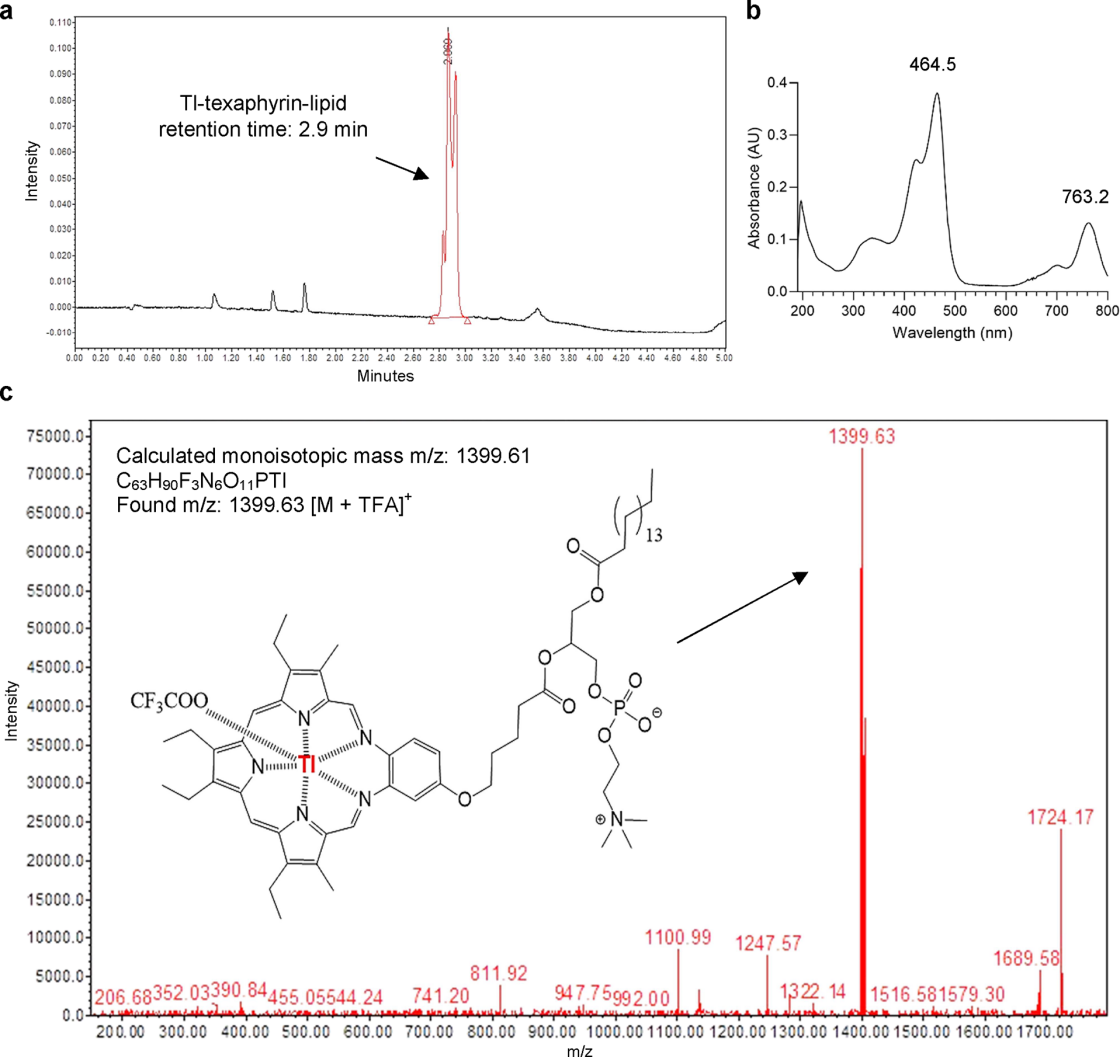
Nonradioactive Tl-texaphyrin-lipid conjugates. (a) Chromatogram of Tl-texaphyrin-lipid (oxidizing agent used) with the elution peak at 2.9 min (465 nm wavelength) and corresponding (b) absorption spectrum and (c) mass spectrum of Tl-texaphyrin-lipid; *m*/*z* found: 1399.63 (main peak) matching formula of C_63_H_91_F_3_N_6_O_11_PTl (protonated Tl-texaphyrin-lipid associated with one trifluoroacetate ion); mobile phase: 0.1% TFA/acetonitrile. Exact mass: 1399.61, *m*/*z* 1399.61 (100%), 1400.61 (63.9%), and 1397.61 (37.7%). Shoulder peaks in the main peak eluting at 2.9 min correspond to phospholipid regioisomers formed during the synthesis of texaphyrin-lipid compounds.

**Figure 3:**
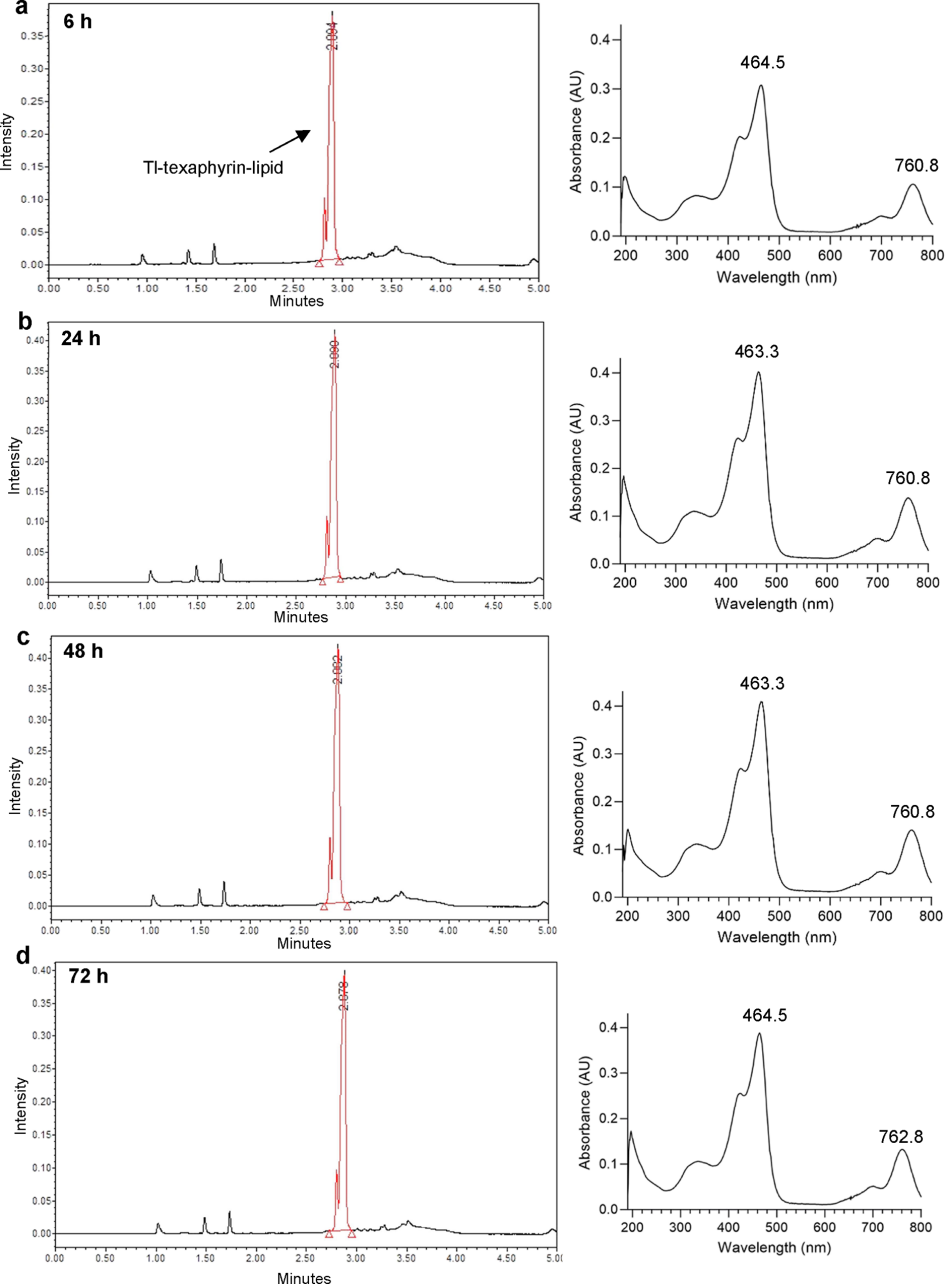
Stability of nonradioactive Tl-texaphyrin-lipid conjugates up to 72 h in ethanol solution. UHPLC-MS chromatograms showing the peak eluting at around 2.9 min characteristic for Tl-texaphyrin-lipids and a corresponding absorption spectrum after (a) 6 h, (b) 24 h, (c) 48 h, and (d) 72 h. Absorption peaks characteristic for metalated texaphyrins are around 465 and 760 nm. Shoulder peaks in the main peak eluting at 2.9 min correspond to phospholipid regioisomers formed during the synthesis of texaphyrin-lipid compounds.

Free-base texaphyrin-lipid compounds (without a metal conjugated to the basic macrocycle) can be susceptible to degradation of the texaphyrin macrocycle under acidic conditions, as the imine can experience rapid acid hydrolysis.^27^ To minimize texaphyrin-lipid macrocycle degradation during Tl^3+^ chelation, it was necessary to reduce the acidity of the solution containing oxidized Tl^3+^. The chelation conditions were first examined with stable TlCl to ensure the formation of Tl-texaphyrin-lipid in high yield. Approximately 20 to 30 min after the addition of oxidized Tl^3+^, visual evidence of a color change from brown to dark green was observed as the Tl^3+^ chelates to form the aromatic metal-texaphyrin complex (Suppl. Figure 3a). Tl-texaphyrin-lipid samples remained stable for an extended duration when oxidized nonradioactive Tl^3+^ was diluted and neutralized with ddH_2_O or NH_4_OAc, as opposed to PBS or ethanol. The formation of the Tl-texaphyrin-lipid complex was confirmed by the characteristic absorption Soret-like and Q-like bands at 465 and 760 nm, respectively (Suppl. Figure 3b,c). Tl^+^ did not form metal-texaphyrin-lipid complexes under the same conditions in the absence of the oxidation agent (Suppl. Figure 3b). Such metal complex absorption spectra have previously been reported for other metal-chelated texaphyrin-lipids.^22^ The absorption spectra of Tl-texaphyrin-lipid samples remained unchanged for up to 72 h at RT (Suppl. Figure 3c), with the consistent optical properties (green color) persisting, which further indicated the chelation stability at RT. However, when the Tl-texaphyrin-lipids were stored at 37 °C for 24 h, the color of the sample turned pink, indicating demetalation of the Tl-texaphyrin-lipid complex and degradation of the macrocycle structure (Suppl. Figure 3d). This degradation was confirmed by UV–Vis spectroscopy, which showed no distinctive peaks at 760 nm characteristic of the Tl-texaphyrin-lipid complex. During the UHPLC-MS analysis, Tl-texaphyrin-lipid exhibited a retention time of 2.9 min with the corresponding molecular mass and absorption ([M + TFA]^+^ 1399.63, λ_Abs_ 465, 763 nm), while unchelated texaphyrin-lipid had a retention time of approximately 3.6 min which corresponded to the free-base texaphyrin-lipid ([M + H]^+^ 1088.17, λ_Abs_ 364 nm) and is in concordance to the retention time of free-base texaphyrin-lipid that has previously reported by Cheng et al. under the same UHPLC-MS conditions (Figure 2a–c and Suppl. Figure 4a).^21^ The addition of Tl^+^ to texaphyrin-lipid solution in the absence of the oxidizing agent (iodination beads) did not show evidence of complex formation (Suppl. Figure 4b). The stability of Tl-texaphyrin-lipid samples kept at RT for up to 72 h was also confirmed by UHPLC-MS analysis (Figure 3).

**Figure 4:**
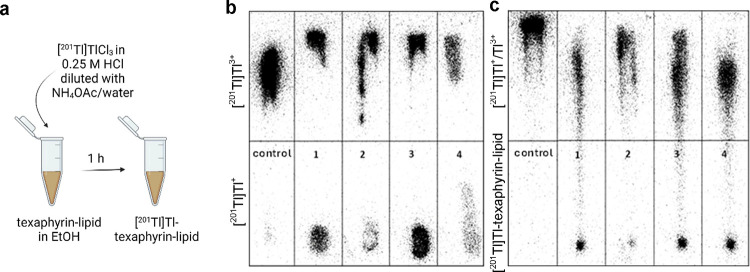
[^201^Tl]Tl-texaphyrin-lipid conjugates. (a) Schematic illustration of [^201^Tl]Tl-texaphyrin-lipid complex formation. (b) iTLC-SG strips showing [^201^Tl]Tl^+^ at the origin (bottom) and [^201^Tl]Tl^3+^ at the solvent front (top) after diluting oxidized [^201^Tl]Tl^3+^ in 0.25 M HCl 5 times with various solutions/buffers (1 to 4). Stationary phase: iTLC-SG, mobile phase: acetone. [^201^Tl]Tl^3+^ in these conditions migrates with the solvent front, while [^201^Tl]Tl^+^ remains at the origin. (c) iTLC-SG strips showing [^201^Tl]Tl-texaphyrin-lipid and free [^201^Tl]Tl^+^**/**Tl^3+^; stationary phase: iTLC-SG, mobile phase: 10% EDTA in 0.1 M NH_4_OAc. [^201^Tl]Tl^+^**/**Tl^3+^ in these conditions migrate with the solvent front, while [^201^Tl]Tl-texaphyrin-lipid remains at the origin. Solvents/buffers used: (1) water, (2) 99.9% ethanol, (3) phosphate-buffered saline, and (4) 0.1 M NH_4_OAc buffer (pH = 7); control: [^201^Tl]Tl^3+^ in 0.25 M HCl.

After confirming the formation of Tl-texaphyrin, we proceeded to radioactive [^201^Tl]Tl^3+^ chelation. The oxidized thallium ([^201^Tl]Tl^3+^) solution in 0.25 M HCl was immediately neutralized with different basic solvents and buffers prior to adding to the texaphyrin-lipid ethanol solution in order to prevent its decomposition observed in acidic conditions (Figure 4a–c). The solutions tested to neutralize acidic conditions included ddH_2_O, 99.9% ethanol, PBS, and 0.1 M NH_4_OAc buffer (pH = 7). The optimal solution was selected based on the quantification of iTLC results for [^201^Tl]Tl^3+^ oxidation and subsequent chelation (Suppl. Table 2). The highest chelation efficiency was observed when [^201^Tl]Tl^3+^ was diluted with either ddH_2_O or 0.1 M NH_4_OAc buffered solution which resulted in the texaphyrin-lipid radiolabeling yields of 25.5 ± 4.5 and 23.3 ± 3.2%, respectively.

### ^nat^Tl-Nanotexaphyrin and [^201^Tl]Tl-Nanotexaphyrin Formulation: Characterization and Uptake in Cancer Cells

**Figure 5:**
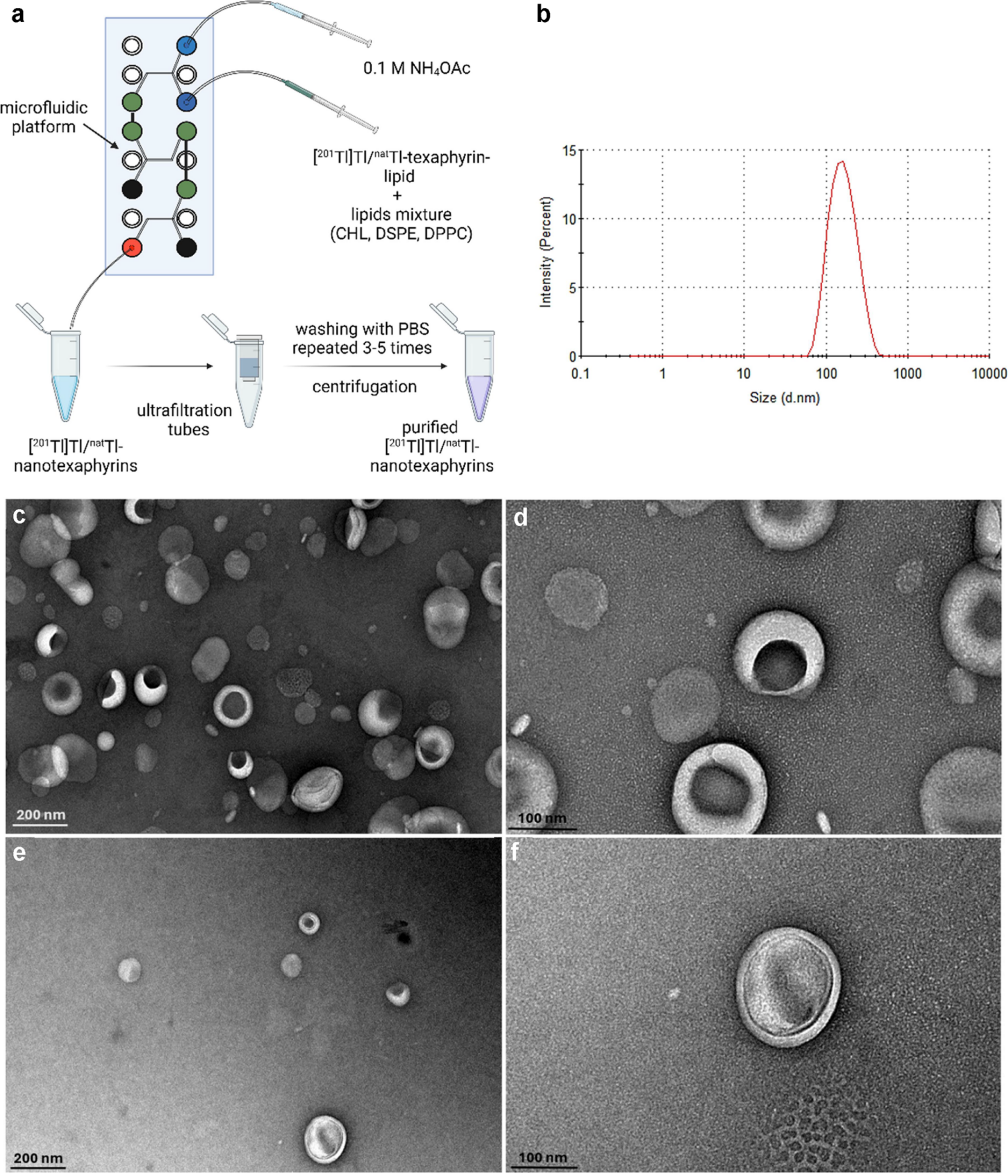
Tl-nanotexaphyrins formation and characterization. (a) Schematic illustration of [^201^Tl]Tl/^nat^Tl-nanotexaphyrin synthesis and purification. (b) Representative DLS analysis of Tl-nanotexaphyrins showing size distribution by intensity. *Z*-average: 152.4 nm; PDI: 0.167. (c–f) TEM images of Tl-nanotexaphyrins in PBS at 80,000 magnification (scale bar: 200 nm) and 200,000 magnifications (scale bar: 100 nm).

After successful chelation of the [^201^Tl]Tl/Tl-texaphyrin-lipid, we validated the self-assembly of the chelated texaphyrin (10–20% of total molar lipid content) by combining with DPPC (34.4–44.4% total molar lipid content), cholesterol (40.6% total molar lipid content), and DSPE-PEG_2000_ (5.0% total molar lipid content) in 99.9% ethanol solution. Subsequently, the ethanol phase containing lipids was rapidly mixed with an aqueous phase of 0.1 M NH_4_OAc buffer, pH 7.0, through a microfluidic system to yield Tl-nanotexaphyrins (Figure 5a). DLS and TEM measurements of Tl-nanotexaphyrins confirmed their homogeneity, with an average hydrodynamic diameter of 147.4 ± 1.4 nm and a PDI of 0.15 ± 0.03 (Figure 5b–f). The measured zeta potential was slightly negative: −1.5 ± 0.4 mV (*n* = 3). Stability assessments of Tl-nanotexaphyrins in PBS over 72 h and across a temperature range of 4–37 °C indicated consistent size and dispersity (Table 1).

**Table 1:** Tl-Nanotexaphyrins Stability Over Time at Various Temperatures^a^

time	0 h		24 h		48 h		72 h	
*T* (°C)	size (nm)	PDI	size (nm)	PDI	size (nm)	PDI	size (nm)	PDI
4	147.4 ± 1.4	0.150	143.2 ± 2.7	0.140	143.7 ± 1.1	0.154	144.2 ± 0.8	0.150
25			143.8 ± 1.8	0.152	144.8 ± 2.0	0.159	144.1 ± 1.0	0.162
37			142.8 ± 1.2	0.151	146.0 ± 1.3	0.193	155.9 ± 3.6	0.230

a Size of nonradioactive Tl-nanotexaphyrins and the PDI was assessed by DLS in PBS from 0 to 72 h at 4, 25, and 37 °C, *n* = 3.

**Figure 6:**
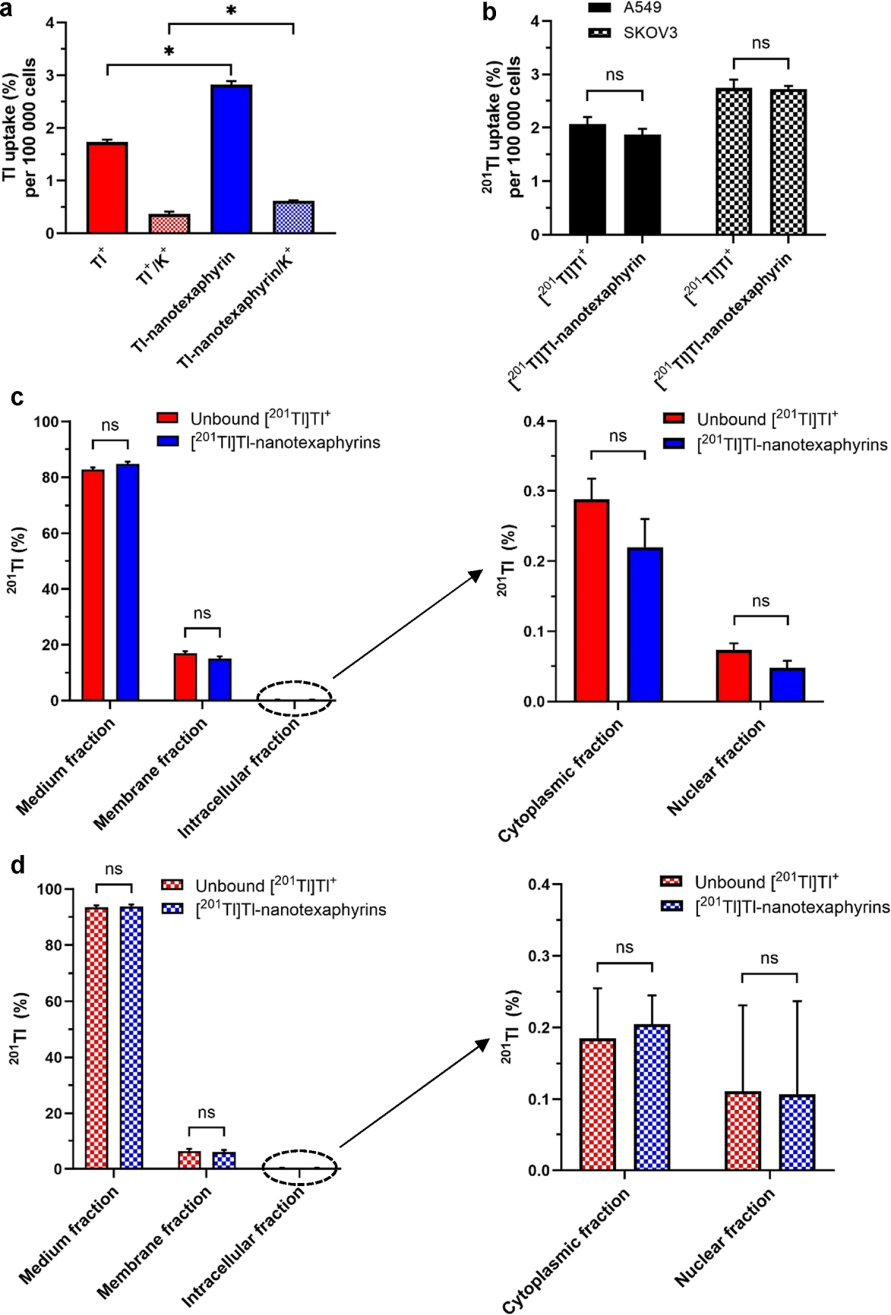
Cellular uptake of Tl-nanotexaphyrins and [^201^Tl]Tl-nanotexaphyrins. (a) Uptake of Tl-nanotexaphyrins, measured by ICP-MS, in lung cancer cells (A549) incubated for 16 h with nonradioactive Tl-nanotexaphyrins. Oxidized TlCl at the same concentration was used as a control. KCl solution (25 mM) was used to inhibit unbound Tl^+^ uptake. 100,000 cells were seeded per well, *p* = 0.0018 and 0.0084. (b) [^201^Tl]Tl-nanotexaphyrin and unbound [^201^Tl]Tl^+^ uptake in lung cancer cells (A549) and ovarian cancer cells (SKOV3) calculated per 100,000 cells. (c) Cell fractionation results in A549 cells per total number of cells counted (665,000) after 16 h incubation with [^201^Tl]Tl-nanotexaphyrins and unbound [^201^Tl]Tl^+^. (d) Fractionation results for SKOV3 cells per total number of cells counted (210,000) after 16 h of incubation with [^201^Tl]Tl-nanotexaphyrins and unbound [^201^Tl]Tl^+^. Bars represent mean ± SD, * indicates significance with *P* < 0.05, paired *t* test, ns indicates not significant.

To characterize the transport of Tl-nanotexaphyrin into cells, we first quantified the cellular uptake of nonradioactive Tl-nanotexaphyrins following a 16 h incubation in lung cancer cells (A549) using ICP-MS. To inhibit unchelated Tl^+^ ion uptake, cells were incubated in 25 mM KCl solution, as high concentrations of K^+^ in medium suppress Tl^+^ uptake in cells due to the competition for Na^+^/K^+^ pump binding sites.^4^ Tl-nanotexaphyrin uptake in A549 cells was 2.8 ± 0.07% (per 100,000 cells), 1.6 times higher (significant, *p* = 0.0018) than the uptake measured for the unbound Tl^+^ control (1.7 ± 0.05%). However, when the Na^+^/K^+^ pump was inhibited by KCl solution, Tl-nanotexaphyrins uptake decreased to 0.6 ± 0.01%, though it remained 1.7 times higher than that of the unbound Tl^+^/K^+^ control (significant, *p* = 0.0084, Figure 6a). After optimizing the formulation of nonradioactive Tl-nanotexaphyrins, we further investigated the formulation of [^201^Tl]Tl-nanotexaphyrin. Using the same lipid ratio and lipid component (cholesterol: 40.6% total molar lipid content, DPPC: 34.4–44.4% total molar lipid content, and DSPE-PEG_2000_: 5.0% total molar lipid content) with [^201^Tl]Tl-texaphyrin-lipids in 99.9% ethanol solution, the same synthetic method as for the nonradioactive Tl-nanotexaphyrin formulation was performed. The progress of [^201^Tl]Tl^3+^ oxidation and radiochemical purity of [^201^Tl]Tl-labeled nanoparticles using the iTLC method was assayed using methods described earlier (Suppl. Figure 2b). The radiolabeling yield of [^201^Tl]Tl-nanotexaphyrins averaged 5.1 ± 4.4% with nanoparticle radiochemical purity of 77.4 ± 10.3% (Table 2, *n* = 4).

**Table 2:** [^201^Tl]Tl-Nanotexaphyrin Synthesis^a^

reaction number	1	2	3	4
[^201^Tl]Tl^+^ to [^201^Tl]Tl^3+^ oxidation yield	90.0%	96.2%	90.2%	85.9%
[^201^Tl]Tl^3+^ dilution solvent/buffer	NH_4_OAc	NH_4_OAc	NH_4_OAc	H_2_O
[^201^Tl]Tl-nanotexaphyrin purity prepurification	16.7%	20.2%	29.9%	24.4%
[^201^Tl]Tl-nanotexaphyrin purity postpurification	71.6%	82.9%	66.1%	88.8%
radiolabeling yield	7.8%	9.8%	2.4%	0.5%

a Results from four independent experiments involving [^201^Tl]Tl^+^/Tl^3+^ oxidation, dilution, and chelation reaction with texaphyrin-lipids, synthesis of [^201^Tl]Tl-nanotexaphyrins, and the purification process. Radiolabeling yield was determined by comparing the activity of purified [^201^Tl]Tl-nanotexaphyrins to the initial activity of [^201^Tl]TlCl stock used for the experiment. [^201^Tl]Tl^+^/Tl^3+^ oxidation yield was assessed by iTLC using acetone as a mobile phase. [^201^Tl]Tl-nanotexaphyrin radiochemical purity before and after the purification process was assessed by iTLC using 10% EDTA in 0.1 M NH_4_OAc as a mobile phase. Data are presented as mean ± SD, *n* = 4.

The capability of [^201^Tl]Tl-nanotexaphyrins to retain ^201^Tl when stored in a PBS solution for various times was assessed using the iTLC method (mobile phase: 10% EDTA in 0.1 M NH_4_OAc) up to 72 h (Table 3). After 72 h of incubation, radiochemical purity of [^201^Tl]Tl-nanotexaphyrins decreased from 77.5 ± 16.0 to 56.0 ± 1.4% at 4 °C and 29.1 ± 7.3% at 37 °C.

**Table 3:** [^**201**^Tl]Tl-Nanotexaphyrin Radiostability in PBS Solution^a^

time	temperature	
	4 °C	37 °C
0 h	77.5 ± 16.0%	77.5 ± 16.0%
2 h		70.7 ± 9.7%
6 h		59.5 ± 13.3%
72 h	56.0 ± 1.4%	29.1 ± 7.3%

a iTLC method was used to assess the radiostability; stationary phase: iTLC-SG, mobile phase: 10% EDTA in 0.1 M NH_4_OAc; [^201^Tl]Tl^+^/Tl^3+^ in these conditions migrate with the solvent front, while [^201^Tl]Tl-texaphyrin-lipids remain at the origin. Data are presented as mean ± SD.

The uptake of [^201^Tl]Tl-nanotexaphyrins in lung cancer cells (A549) and ovarian cancer cells (SKOV3) after 16 h of incubation at 37 °C was found to be 2.2 ± 0.11 and 2.7 ± 0.08%, respectively (Figure 6b). These uptake values closely resemble those found for the unbound ^201^Tl uptake (2.5 ± 0.30% in A549 cells and 2.9 ± 0.06% in SKOV3 cells).

Cell fractionation performed on A549 cells and SKOV3 cells following incubation with [^201^Tl]Tl-nanotexaphyrins or unbound [^201^Tl]Tl^+^ revealed that the amount of intracellular activity was significantly lower compared to the activity collected with the “membrane” fraction (Figure 6c,d), likely due to a rapid efflux of unbound [^201^Tl]Tl^+^ during subsequent cell washing steps in the fractionation procedure.^4^^201^Tl activity distribution in cells incubated with [^201^Tl]Tl-nanotexaphyrins remained similar to cells incubated with unbound [^201^Tl]Tl^+^ in both cell lines.

## Discussion

Despite its high radiotoxic potential due to Auger electron emissions and its past widespread availability due to previous use in myocardial imaging, ^201^Tl has not yet been fully evaluated for targeted cancer therapy. This is largely because of the lack of suitable chelators for complexing ^201^Tl to ligands that could be targeted to cancer cells. Texaphyrins, which contain a large pentadentate macrocycle, are able to form stable complexes with a wide range of trivalent metals such as In^3+^, Lu^3+^, and Bi^3+^,^18^ and hence potentially presented a good opportunity to efficiently bind radioactive thallium.

In this study, we synthesized nonradioactive and radioactive Tl-texaphyrin-lipid compounds and investigated their stability and self-assembly into nanoparticles, with the primary aim to transport radioactive thallium into cancer cells for radiotherapeutic purposes. Using nonradioactive thallium salts, Tl-texaphyrin-lipid compounds were synthesized, characterized, and proved to be stable when kept at 25 °C in ethanol solution for a minimum of 72 h. To the best of our knowledge, these types of complexes have not been previously described in the literature. Furthermore, the amphipathic nature of the texaphyrin phospholipids enabled the Tl-texaphyrin-lipids to self-assemble into homogeneous and spherical liposome-like structures around 150 nm in diameter, which were stable in aqueous conditions. No significant alterations in the size or dispersity of Tl-texaphyrin nanoparticles were detected by DLS, when measured up to 72 h at RT and 37 °C. A microfluidic system was used in the preparation of Tl-texaphyrin nanoparticles to induce laminar flow, enabling rapid mixing of organic and aqueous phases, which in turn facilitated the formation of homogeneous, controlled size liposomes.^21^ This method is simple and versatile for nanoparticle formation, eliminating the need for more labor-intensive techniques such as extrusion. Furthermore, when applied to the preparation of radioactive [^201^Tl]Tl-nanotexaphyrins, it minimizes the radiation exposure and reduces the risk of cross-contamination with radioactivity. In this study, for the first time, we synthesized ^201^Tl-texaphyrin-lipid and evaluated their biological stability *in vitro*. During our experiments, we noticed that increased temperature (37 °C or above) had a negative impact on formation and stability of Tl-texaphyrin-lipid compounds. Although the change in temperature did not influence [^201^Tl]Tl^+^ oxidation in acidic conditions, [^201^Tl]Tl^3+^ ions were likely dissociated from the texaphyrin macrocycle due to Tl^3+^ reduction to the 1+ oxidation state in neutralized aqueous conditions, especially at physiological temperatures, following a mechanism that is not yet understood. Despite the instability of these complexes, the findings of this study offer valuable insights into the potential of texaphyrins as chelators for radioactive thallium.

The multiple functionalization sites available on the texaphyrin ring allow for the addition of various moieties, enabling specific functionalities and potentially enhancing the stability of the metal complex. Modifying the texaphyrin-lipid structure by adding supplementary groups containing electron-rich atoms, such as carboxyl groups or amide groups, might prevent Tl reduction to the 1+ oxidation state by providing additional axial ligands and thereby increase complex stability. It has been shown previously that metals like Mn, In, Gd, and Lu can form stable complexes with texaphyrins in physiological conditions.^19,21,23,28^ Mn with a 2+ oxidation state was found to form Mn-texaphyrin-lipid complexes with one axial ligand, while texaphyrin-lipid conjugates with trivalent metals (In^3+^, Lu^3+^, and Gd^3+^) possessed two axial ligands. However, Tl-texaphyrin-lipid complexes with only one axial ligand were detected using UHPLC-MS. The stability of the texaphyrin-lipid complex with Tl^3+^ could potentially be further increased either by altering the counteranion acting as an axial ligand or by integrating additional ligand moieties into the texaphyrin structure.

It was also observed that Tl^+^ binding by the texaphyrin-lipids does not occur in any tested conditions. The increased ionic radius of Tl^+^ in comparison to Tl^3+^ (164 pm vs 103 pm)^7^ and the greater polarizability significantly enhance the "softness" of its electron-acceptor character with a stronger affinity for donors like sulfur.^29^ Therefore, it is conceivable that further expansion of the porphyrinoid macrocycle to accommodate a larger metal cation or attaching additional sulfhydryl groups could enable ^201^Tl^+^ complexation without the need for oxidation.^30−32^

In nanotexaphyrins, the texaphyrin macrocycle is expected to reside in the hydrophobic leaflet of the bilayer membrane, remaining unaffected by the surrounding aqueous environment and any potential reducing agents present in the culture medium or cell cytoplasm. Nevertheless, [^201^Tl]Tl^3+^ complexation within the nanotexaphyrin structure proved unstable, and ^201^Tl was released after 72 h and nearly twice as fast at 37 °C than at RT when kept in PBS. This suggests that [^201^Tl]Tl-nanotexaphyrin’s liposomal-like nanostructure did not provide additional protection against the reduction of Tl^3+^ to Tl^+^ at 37 °C. In cellular uptake experiments with radioactive Tl, as well as in ^201^Tl subcellular distribution experiment, the results for unbound [^201^Tl]Tl^+^ and [^201^Tl]Tl-nanotexaphyrins were very similar, suggesting the reduction of [^201^Tl]Tl^3+^ to [^201^Tl]Tl^+^, and the demetalation from the texaphyrin ring under the conditions tested. This pattern of behavior in the *in vitro* experiments has been previously observed for other types of thallium chelators^8^ and indicates that ^201^Tl^3+^ ions were likely reduced to ^201^Tl^+^ and subsequently released under experimental conditions, either already within the medium or intracellularly following uptake of Tl-nanotexaphyrins. This process may have been intensified by the presence of reducing agents. Glutathione, a tripeptide-like molecule containing thiol, is abundant in the cytoplasm of eukaryotic cells, where it plays a crucial role in cellular redox homeostasis as the primary low-molecular weight reducing agent.^33^ Glutathione is also present in the culture medium. Its reduced form could facilitate the reduction of Tl^3+^ to Tl^+^ upon oxidation to glutathione disulfide.^34^ Furthermore, the negative effect of acidic conditions, such as those present inside lysosomes, may contribute to thallium instability. As Tl^+^ cannot be easily bound by the texaphyrin macrocycle due to its larger radius, it is released from the Tl-texaphyrin-lipid complex. Vulnerability of Tl(III) to reduction is a continuing problem for efforts to design thallium(III) chelators, and therefore, more experiments are needed to fully investigate Tl-nanotexaphyrin stability under reducing conditions, exposed to acidic environments, and at increasing temperatures. Substituting the neutral buffer (0.1 M NH_4_OAc) with a oxidizing buffer like Mn(OAc)_3_ during the formulation process could influence the maintenance of thallium in a higher oxidation state within the texaphyrin macrocycle.^35^

It is worth noting that the slow intracellular release of [^201^Tl]Tl^+^ from nanoparticles may prove beneficial in some circumstances, especially when [^201^Tl]Tl-nanotexaphyrin conjugates are locally-delivered to a tumor (e.g., intratumoral delivery). Auger electrons released during ^201^Tl radioactive decay have a very short range (<1 μm), and it is likely that they have to be present in the close proximity of the radiosensitive cellular targets, such as the nucleus or the cell membrane.^36,37^ Enabling a gradual release of [^201^Tl]Tl^+^ ions from nanoparticles once inside the cell and their diffusion in the cytosol could avoid the need for additional subcellular targeting of the nanostructures in order to achieve maximum radiotoxic impact.

Translating the optimized, nonradioactive method of synthesizing Tl-nanotexaphyrins to bind radioactive ^201^Tl proved difficult. Despite successful chelation and formulation of radioactive [^201^Tl]Tl-nanotexaphyrins, with an average purity of 77.4 ± 10.3% after the purification process, the radiolabeling yield of [^201^Tl]Tl-nanotexaphyrins was low (5.1 ± 4.4%), and this severely limited the scalability of the reaction to produce enough activity for further studies. All our attempts to further optimize the chelation reaction and nanoparticle synthesis to increase the radiolabeling yield, including “doping” [^201^Tl]Tl-nanotexaphyrins with nonradioactive Tl or combining ^201^Tl with Cd-texaphyrin-lipid or Mn-texaphyrin-lipids using the postinsertion method,^22^ proved unsuccessful. The difference between the nonradioactive and radioactive Tl-texaphyrin-lipid chelation rate and stability could be linked to a concentration of Tl present in the reaction solution, which would be around 1000–2000 times lower in [^201^Tl]TlCl_3_ (^201^Tl and ^203^Tl combined) than TlCl_3_ used in nonradioactive experiments. The difference could be related to the competition between Tl^3+^ reduction to the 1+ oxidation state and its complexation with the texaphyrin. With a minimal amount of ^201^Tl, most of it will be reduced by even trace levels of adventitious reducing agents, leaving only a small portion of thallium to complex with texaphyrins. Conversely, when there is a higher concentration of Tl, more Tl^3+^ is available to bind with texaphyrins. It is possible that the structure of texaphyrin stabilizes Tl^3+^, which could account for the higher incorporation yield observed with nonradioactive Tl. This suggests that the texaphyrin macrocycle might shield Tl^3+^ from reduction, albeit temporarily.

The stabilizing effect of higher thallium concentration was evident in the cellular uptake experiments, where the uptake of nonradioactive thallium bound to nanotexaphyrin was significantly higher than that of unbound thallium, both with and without KCl. However, it was clear that the uptake of Tl-nanotexaphyrins is influenced by potassium ions in a manner similar to that of unbound Tl^+^. This suggests that thallium bound to nanotexaphyrins taken up by cells may subsequently be released from the macrocyclic complex and exported from the cells as Tl^+^. The reuptake of Tl^+^ exported by the cells could then be inhibited by higher concentrations of potassium ions, which compete for binding sites on the sodium–potassium pump.

Another reason for the low radiolabeling rate of texaphyrin-lipids with Tl^3+^ is the incompatibility of the oxidation conditions with stability of texaphyrin-lipid compounds. Tl^3+^ in a form that is readily available for complexation appears to be more stable in acidic conditions; in contrast, a concentrated acidic environment led to rapid degradation to texaphyrin-lipids.^27^ Neutralizing the acidic conditions to reduce the macrocycle degradation rate substantially reduced the amount of Tl^3+^ present in the sample by enhancing the Tl^3+^ to Tl^+^ reduction process. The low radiolabeling rate is also due to a retention of [^201^Tl]Tl^3+^ on the oxidation bead surface during the oxidation process (30–50% of ^201^Tl activity), and further improvements to the oxidation and chelation conditions are needed in order to increase ^201^Tl radiolabeling efficiency.

Additional research is necessary to thoroughly assess the stability and radiotoxicity of [^201^Tl]Tl-nanotexaphyrins. Enhancing the thermodynamic stability of [^201^Tl]Tl-nanotexaphyrins could involve various approaches, such as chemically modifying the nanotexaphyrin structure with additional coordination groups, expanding the texaphyrin ring, or utilizing an extended porphyrin geometry to accommodate large Tl ions. Furthermore, employing different nanotexaphyrin formulation strategies, including various buffers and lipid compositions, as well as modifying nanoparticle surfaces, could be effective in reducing the rate of Tl^+^ release from the metal complex.

Although the presented strategy for synthesizing [^201^Tl]Tl-nanotexaphyrins is suboptimal, it is worth considering the use of texaphyrins alone as chelators for ^201^Tl, omitting the fragile lipid component. Further modifications to the texaphyrin structure to enhance stability, along with attaching targeting peptides such as PSMA, could potentially prove successful for targeted Auger electron therapy with radiothallium.

## Conclusions

In this study, we successfully synthesized and characterized the first Tl(III)-texaphyrin-lipid complexes, demonstrating their chelation stability at room temperature for at least 72 h in ethanol solution. These Tl-texaphyrin-lipids were capable of self-assembly into homogeneous Tl-nanotexaphyrins of around 150 nm in size, which remained stable under aqueous conditions. The synthesis of [^201^Tl]Tl-nanotexaphyrins achieved reasonable radiochemical purity (around 77%), albeit with low radiolabeling efficiency (around 5–10%). However, [^201^Tl]Tl^3+^ reduction and subsequent release of [^201^Tl]Tl^+^ ions from [^201^Tl]Tl-nanotexaphyrins under biological conditions (culture medium, 37 °C) render these complexes unstable and unsuitable for delivering radioactive ^201^Tl without further modifications. Nevertheless, this study provides valuable insights into radiometal chelates with rapid redox properties and offers a better understanding of the future development of ^201^Tl chelators. The chelation of ^201^Tl by texaphyrins is promising for the design of other multidentate macrocyclic ligands that could be incorporated into delivery vehicles for targeted tumor delivery.

## Supporting Information Available

Additional experimental details and methods, including photographs of experimental results, UV–Vis, and UHPLC-MS results (PDF)
