# Supplementary material for: Synthesis and Characterization of Thallium-Texaphyrin Nanoparticles and Their Assessment as Potential Delivery Systems for Auger Electron-Emitting 201Tl to Cancer Cells
Source: Mol Pharm. 2024 Dec 16;22(1):242–54. doi: 10.1021/acs.molpharmaceut.4c00873 (PMC11707725; doi:10.1021/acs.molpharmaceut.4c00873)
Supplement: Supplementary file 1 — mp4c00873_si_001.pdf [file mp4c00873_si_001.pdf]

Supporting Information

Synthesis and Characterisation of Thallium-Texaphyrin Nanoparticles and Their Assessment as Potential Delivery Systems for Auger Electron-Emitting ^{201}Tl to Cancer Cells

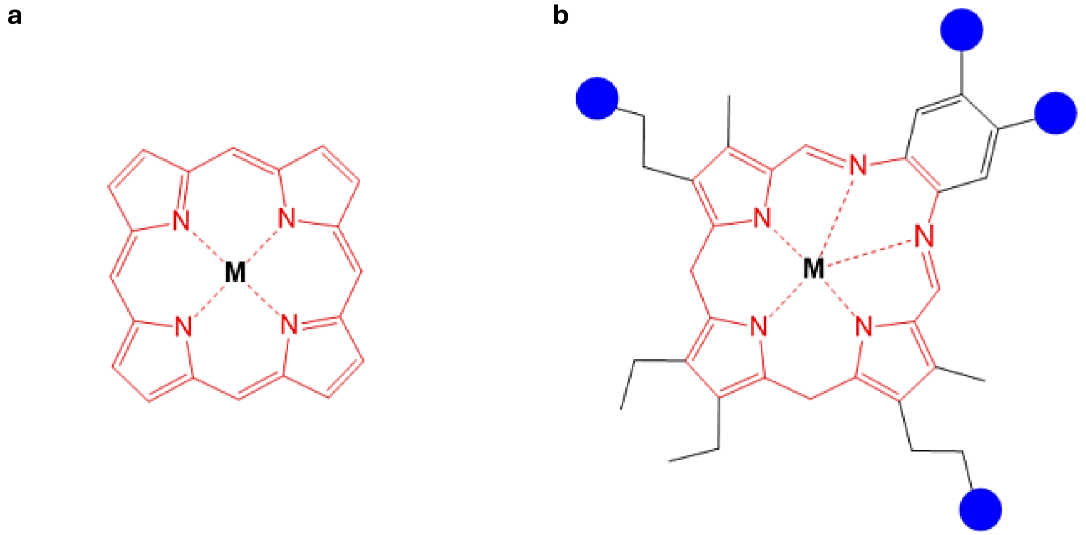

Suppl. Figure 1. Comparison of porphyrin and texaphyrin macrocycles. (a) The structure of porphyrin macrocycle with coordinated metal ion. (b) The structure of texaphyrin with macrocycle marked in red and sites available for functionalisation (blue dots). M - coordinated metal. Created with ChemDraw.

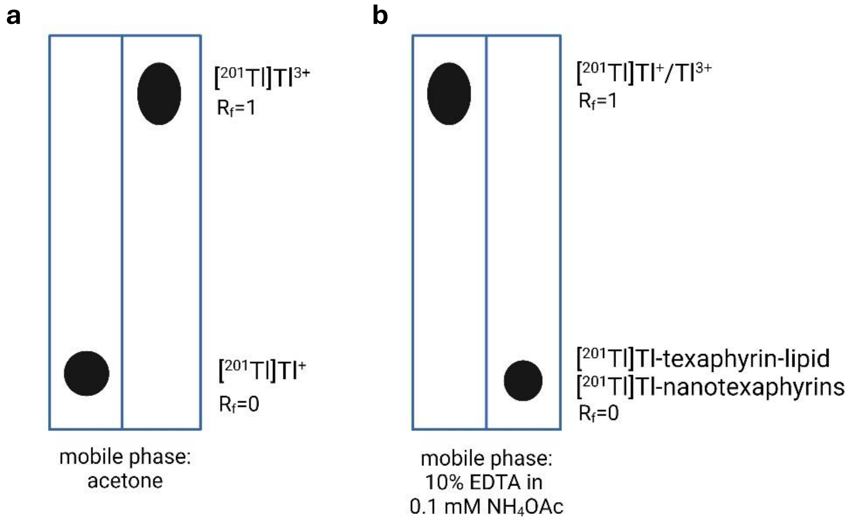

Suppl. Figure 2. Schematic representation of the iTLC method used in the study. iTLC plate showing (a) quantification of ^{201}Tl oxidation reaction and (b) assessing [^{201}Tl]Tl-texaphyrin-lipid chelation yield and [^{201}Tl]Tl-nanotexaphyrin purity. Stationary phase: iTLC-SG chromatography paper (silica gel impregnated glass fibre, Pall Corporation). Created with BioRender.com.

Suppl. Table 1. [^{201}Tl]Tl^{3+} stability

time	[^{201}Tl]Tl^{3+}
2 h	92.1 ± 3.3%
24 h	91.5 ± 10.6%
48 h	85.8 ± 3.4%
72 h	86.8 ± 2.7%

[^{201}Tl]Tl^{3+} stability in 0.25 M HCl after removal of the oxidation bead is expressed as % of total activity at 4°C. The stability was assessed by the iTLC method, stationary phase: iTLC-SG, mobile phase: acetone, n = 3.

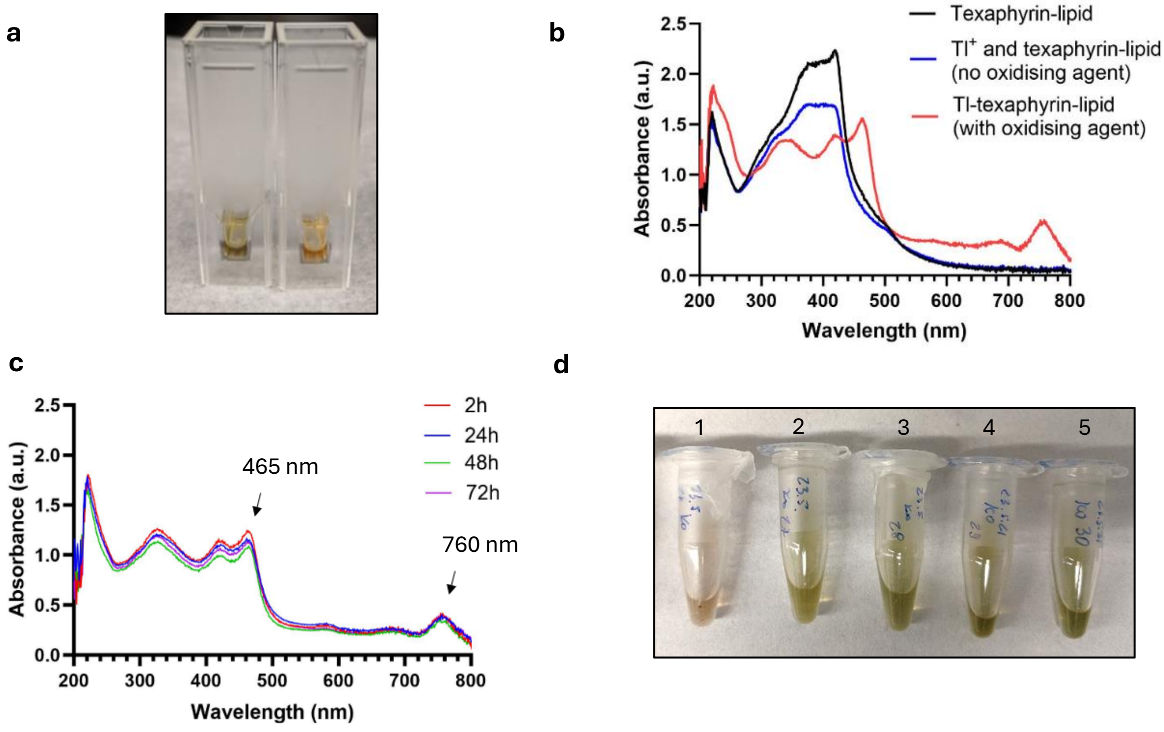

Suppl. Figure 3. Tl-texaphyrin-lipid complexes and their stability. (a) Cuvettes containing a green solution of Tl^{3+}-texaphyrin-lipid (left) and a light brown solution containing non-complexed Tl^{+} with texaphyrin-lipids (right), where oxidising agent was not added. (b) UV-vis spectra of texaphyrin-lipid (black line), Tl-texaphyrin-lipid after oxidation (red line) and Tl^{+} mixed with texaphyrin-lipid without the oxidising agent (blue line). (c) UV-vis spectra of Tl-texaphyrin-lipid (Tl^{+} oxidised with the oxidation bead) after 2-72 h at room temperature (RT). Arrows mark the absorption peaks characteristic for metalated texaphyrins. (d) An image of Tl^{3+}-texaphyrin-lipid samples 24 h after adding Tl^{3+} to texaphyrin-lipid ethanol solution. All samples are green with the exception of sample 1, which was kept for 24 h at 37°C, whereas the rest of the samples (2-5) were kept at 25°C.

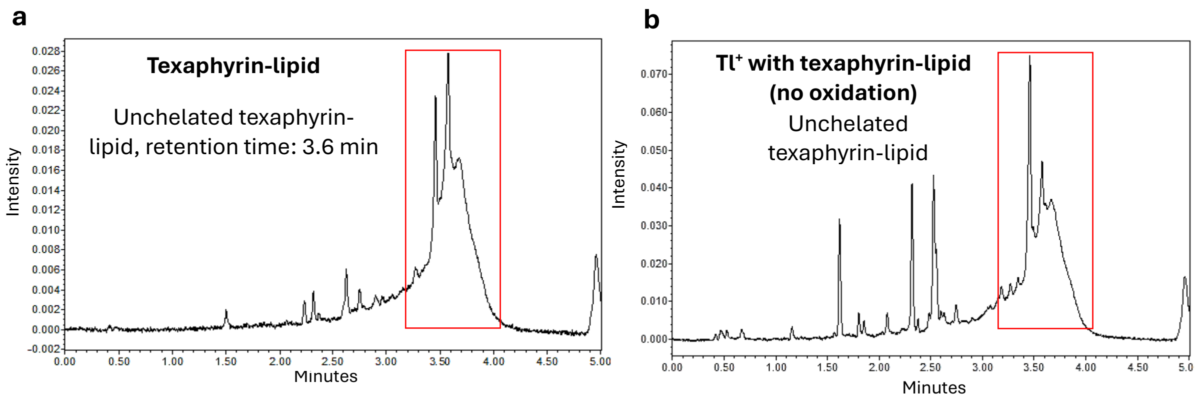

Suppl. Figure 4. UHPLC-MS analysis of Tl-texaphyrin-lipid complexes. (a) Chromatogram of unchelated texaphyrin-lipid showing the elution peak at approximately t_R = 3.6 min with monitoring at 465 nm wavelength. (b) Chromatogram of texaphyrin-lipid mixed with Tl^+ without the oxidising agent monitored at 465 nm wavelength, showing no evidence of complex formation. Complexation of Tl^{3+} by texaphyrin-lipids results in a shift of the t_R to 2.9 mins and absorbance at 465 nm wavelength. Shoulder peaks in the main peak eluting at 3.6 min correspond to phospholipid regioisomers formed during the synthesis of texaphyrin-lipid compounds.

Suppl. Table 2. Optimising conditions of $[^{201}Tl]Tl^{3+}$ chelation to texaphyrin-lipid compounds

Added solution	pH	$[^{201}Tl]Tl^{3+}$	$[^{201}Tl]$Tl-texaphyrin-lipid
1. ddH_2O	1	75.3 ± 6.8%	25.5 ± 4.5%
2. ethanol	1	85.2 ± 2.1%	9.8 ± 2.2%
3. PBS	3	60.1 ± 5.7%	17.6 ± 3.0%
4. NH_4OAc	5	56.8 ± 5.4%	23.3 ± 3.2%

Different solutions (1-4) were added in order to neutralise acidic solution containing $[^{201}Tl]Tl^{3+}$. pH was measured with a pH paper (pHydrion, Micro Essential Laboratory). The amount of $[^{201}Tl]Tl^{3+}$ (%) was quantified by iTLC method using acetone as a mobile phase ($[^{201}Tl]Tl^{+}$: origin; $[^{201}Tl]Tl^{3+}$: solvent front). The amount of $[^{201}Tl]$Tl-texaphyrin-lipid (%) was quantified by iTLC using 10% EDTA solution in 0.1 M NH_4OAc as a mobile phase ($[^{201}Tl]$Tl-texaphyrin-lipid: origin; $[^{201}Tl]Tl^{+}/Tl^{3+}$: solvent front), n = 3.
